# An Alkaloid from Marine *Sirastachys pandanicola* Inhibiting Na^+^-K^+^-ATPase and Ca^2+^-Mg^2+^-ATPase Activity

**DOI:** 10.3390/ph19071127

**Published:** 2026-07-21

**Authors:** Yang Man, Zihao Wang, Boyu Chen, Xiaozhen Diao, Hideo Kigoshi, Yiwen Zhao, Jeevithan Elango, Ahsan Javed, Wenhui Wu

**Affiliations:** 1 Department of Marine Pharmacology, College of Food Science and Technology, Shanghai Ocean University, Shanghai 201306, China; 13500420635@163.com (Y.M.); m240401107@st.shou.edu.cn (Z.W.); 19585290630@163.com (B.C.); xzdiao@shou.edu.cn (X.D.); jelango@ucam.edu (J.E.); 2 Alliance for the Research on the Mediterranean and North Africa (ARENA), University of Tsukuba, Tsukuba 305-8572, Japan; kigoshi@chem.tsukuba.ac.jp; 3 Innovation Research Institute of Traditional Chinese Medicine, Shanghai University of Traditional Chinese Medicine, Shanghai 201203, China; zhaoyw@shutcm.edu.cn; 4 Department of Biomaterials Engineering, Faculty of Health Sciences, UCAM-Universidad Católica San Antonio de Murcia, Campus de los Jerónimos, 135, Guadalupe, 30107 Murcia, Spain; 5 Department of Food Science and Biotechnology, Graduate School, Kyungpook National University, Daegu 41566, Republic of Korea; 6 Marine Biomedical Science and Technology Innovation Platform of Lin-Gang Special Area, Shanghai 201306, China; 7 Putuo Branch of International Combined Research Center for Marine Biological Sciences, Zhoushan 316104, China

**Keywords:** Na^+^-K^+^-ATPase, Ca^2+^-Mg^2+^-ATPase, NMR, marine fungal secondary metabolites

## Abstract

**Background/Objectives**: Marine microorganism metabolites are structurally unique secondary metabolites possessing therapeutic potential. The current study aims to identify a novel ATPase regulator using a newly established bidirectional activity evaluation system to screen for microbial metabolites that inhibit the activities of Na^+^-K^+^-ATPase or Ca^2+^-Mg^2+^-ATPase. **Methods**: A total of 1258 marine microbial strains were isolated from sea mud in Zhoushan, Zhejiang. **Results**: The extract of strain ZSDH2536 exhibited Na^+^-K^+^ and Ca^2+^-Mg^2+^-ATPase inhibitory activity and was identified as *Sirastachys pandanicola* based on morphological and molecular phylogenetic analyses. The secondary metabolite was tentatively identified in the ZSDH2536 strain as a bisindole compound, and named Pandanicoline based on ^1^H-NMR, ^13^C-NMR and high-resolution mass spectrometry analysis. The chemical formula of Pandanicoline is C_51_H_68_N_2_O_10_, with an isotopic mass of 868.4874 Da. The maximum inhibition rate of Pandanicoline on Na^+^-K^+^ and Ca^2+^-Mg^2+^-ATPase was 36.37% and 37.27%, respectively. Moreover, in silico analysis also showed the binding energy of Pandanicoline with Na^+^-K^+^-ATPase was −9.124 kcal/mol and with the Ca^2+^-Mg^2+^-ATPase complex was −10.47 kcal/mol. **Conclusions**: The strain ZSDH2536 represents a promising source of dual inhibitors targeting Na^+^-K^+^ and Ca^2+^-Mg^2+^-ATPase. Pandanicoline exhibits potential as a lead compound for regulating ion homeostasis, providing new opportunities for further investigation into its mechanism and therapeutic applications.

## 1. Introduction

Marine ecosystems constitute approximately half of the world’s total biodiversity and a vast, underexplored reservoir of bioactive compounds with significant potential for functional food development [1]. The unique physicochemical conditions of marine environments, including variations in salinity, temperature, and light, promote the biosynthesis of structurally distinct and biologically active compounds [2]. Marine microorganisms are abundant sources of secondary metabolites such as alkaloids, terpenoids, polyketides, isoprenoids, nonisoprenoids, and quinines [3]. These metabolites often demonstrate unique substitution patterns and physicochemical characteristics, making them a promising yet underexplored source of enzyme-targeting compounds with therapeutic potential.

Natural products have historically served as a foundation for ATPase-targeted drug discovery, offering structurally complex scaffolds capable of fine-tuned enzyme modulation [4]. The Na^+^-K^+^-ATPase maintains transmembrane sodium and potassium gradients that are essential for membrane potential, electrical excitability, and secondary active transport, whereas Ca^2+^-Mg^2+^-ATPase regulates intracellular calcium levels critical for excitation–contraction coupling, vesicular trafficking, and enzymatic signaling cascades [5]. Dysregulation or change in ATPase structures has been implicated in cardiovascular diseases, neurological disorders, and metabolic dysfunctions, highlighting their importance as pharmacological targets. Although cardiac glycosides such as digoxin and verapamil are well-established Na^+^-K^+^-ATPase and Ca^2+^-Mg^2+^-ATPase inhibitors, respectively [6], the clinical application of these drugs often comes with limitations due to toxicity and fewer therapeutic effects. This limitation highlights the need for alternative ATPase regulators with improved selectivity and safety profiles. Recent studies suggest that marine fungal metabolites can regulate different signaling mechanisms against numerous ailments, including cancer, inflammation, diabetes, and also ion transport systems [7]. However, systematic investigations connecting marine fungal secondary metabolites to the dual modulation of Na^+^-K^+^-ATPase and Ca^2+^-Mg^2+^-ATPase remain rare, and the structural identification of bioactive compounds is also difficult due to the unavailability of certain advanced equipment and standards for the structural matching process [8].

Keeping this view in mind, the current trial was conducted to determine the marine fungi strain capable of regulating the Na^+^-K^+^-ATPase and Ca^2+^-Mg^2+^-ATPase activities. Furthermore, strains are taxonomically identified based on molecular phylogenetic analysis and ultrastructural characterization [9]. Bioactivity-guided fractionation combined with HPLC, high-resolution mass spectrometry (LC–MS/MS), and NMR spectroscopy led to the identification of bioactive compounds that regulate the activities of Na^+^-K^+^-ATPase and Ca^2+^-Mg^2+^-ATPase using in vitro and in silico analysis. Conclusively, this study develops the chemical space of marine fungal metabolites and highlights a structurally defined metabolite attenuating ATPase activity with potential relevance for ion transport regulation.

## 2. Results

### 2.1. Morphology and Na^+^-K^+^-ATPase and Ca^2+^-Mg^2+^-ATPase Activity

**Figure 1:**
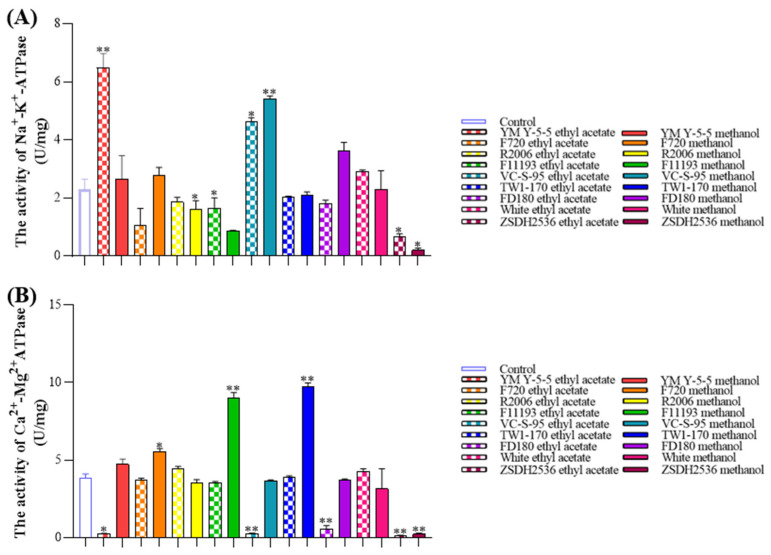
Effect of microbial ethyl acetate and methanol extracts on the enzymatic activities of Na^+^-K^+^-ATPase and Ca^2+^-Mg^2+^-ATPase. (**A**) Na^+^-K^+^-ATPase activity. (**B**) Ca^2+^-Mg^2+^-ATPase activity of nine strains extracted by ethyl acetate and methanol. Comparisons among groups were carried out by one-way analysis of variance (ANOVA). Data are presented as mean ± standard deviation (SD). Each experimental condition was tested in at least three independent replicates (*n* = 3). Error bars in all graphs represent the standard deviation (SD). Values of * (*p* < 0.05); and ** (*p* < 0.01) were considered statistically significant.

Sodium–potassium adenosine triphosphatase Na^+^-K^+^-ATPase and Ca^2+^-Mg^2+^-ATPase maintain transmembrane Na^+^-K^+^ and Ca^2+^-Mg^2+^ gradients through ATP hydrolysis. These electrochemical gradients are fundamental for regulating cell volume, membrane potential, excitation–contraction coupling, vesicular trafficking, and vascular smooth muscle tone [10,11]. Na^+^-K^+^-ATPase and Ca^2+^-Mg^2+^-ATPase are crucial for maintaining cellular homeostasis. Among 1258 microbial strains, 9 marine fungal strains were initially selected and cultured on different media, as depicted in Table 1. After culturing, microbial strains were used to screen the Na^+^-K^+^-ATPase and Ca^2+^-Mg^2+^-ATPase inhibition activity in rat liver tissue. Figure 1A demonstrates that ethyl acetate and methanol extracts of microbial strains R2006, F11193, and TW1-170, along with ZSDH2536, significantly attenuate Na^+^-K^+^-ATPase inhibition. Likewise, microbial strain ZSDH2536 inhibits the Ca^2+^-Mg^2+^-ATPase activity (Figure 1B); in contrast, all the other strains enhance Na^+^-K^+^-ATPase and Ca^2+^-Mg^2+^-ATPase activity. According to the literature, marine fungi contain numerous bioactive compounds (especially polyketides, alkaloids, flavonoids, etc.) that regulate Na^+^-K^+^-ATPase and Ca^2+^-Mg^2+^-ATPase activity [12]. As both fractions of ZSDH2536 substantially inhibit the activity of ATPase, the ZSDH2536 strain was selected for further experiments.

### 2.2. Morphological Characteristics of Strain ZSDH2536

**Figure 2:**
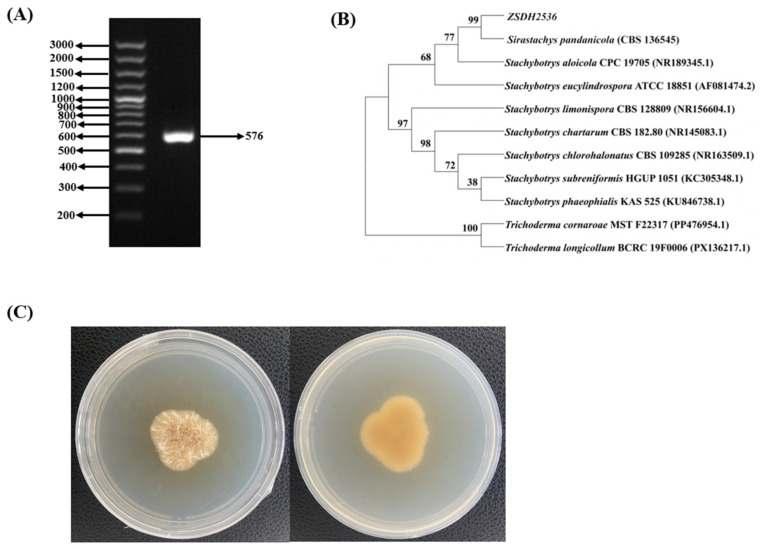
Morphological characterization of strain ZSDH2536. (**A**) PCR product lectropherogram of rDNA ITS of marine microorganism ZSDH2536; (**B**) phylogenetic tree of marine microorganism ZSDH2536; (**C**) actual morphology of the marine microorganism ZSDH2536.

Based on the alignment of 18S rRNA gene sequences. The taxonomic information of strain ZSDH2536 was determined (Figure 2A,B). The sequence of the marine microorganism ZSDH2536 generated a 576 bp nucleotide sequence. The sequencing results were analyzed using the NCBI BLAST website (BLAST+ 2.16.0). The sequences were aligned using the MEGA 12.0.14 software, and a molecular phylogenetic tree was constructed. A phylogenetic tree for the target strain ZSDH2536 was constructed using the neighbor-joining (NJ) method, with the *p*-distance algorithm model applied to the distance tree. The reliability of the NJ phylogenetic tree branches was assessed using bootstrap analysis. The construction of the phylogenetic tree ultimately revealed that the target strain ZSDH2536 is most closely related to *Sirastachys pandanicola*.

**Figure 3:**
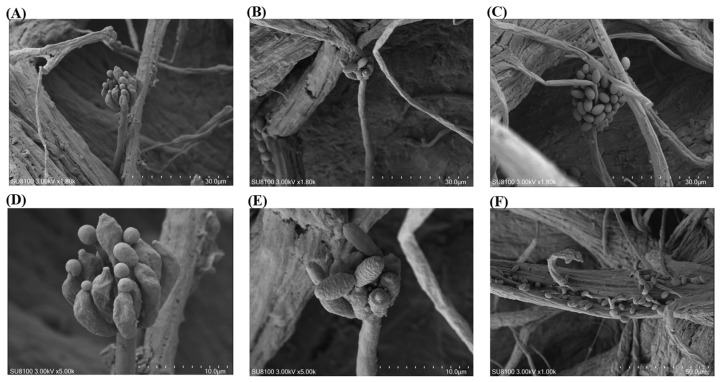
SEM of marine microorganism ZSDH2536. (**A**–**F**) Representation of marine fungi with different SEM camera resolutions.

After cultivation on PDA media for 7–10 days at 28 °C, it developed mature sporulating structures, including a spherical apothecium with comparatively smooth conidiophores (Figure 3A–C). Vegetative growth was characterized by numerous nutrient-absorbing hyphae, which were predominantly slender, sparsely branched, and displayed a creeping growth habit (Figure 3F). These spore-producing cells are pear-shaped and typically arranged in whorls at the tips of the spore stalks. At the open tip of each spore stalk, conidia are continuously produced through internal budding; the mature spores are kidney-shaped or oval with a rough surface and, due to the mucilage on their surface, aggregate into a moist, grape-like spore mass (Figure 3C,D,F). Overall, the conidiophores serve as a supporting framework, the spore-producing cells at the tips act as production units, and conidia—which function as reproductive units—are continuously produced. Based on its morphological characteristics and the results of physiological and biochemical analyses, it was conclusively identified as *Sirastachys pandanicola*.

### 2.3. Isolation, Purification, and Structural Analysis of Compounds

**Figure 4:**
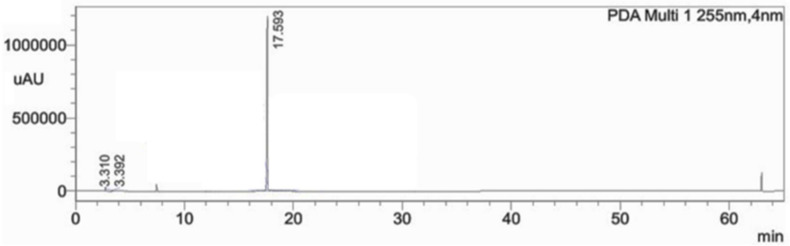
HPLC chromatogram analysis of SP34.

**Figure 5:**
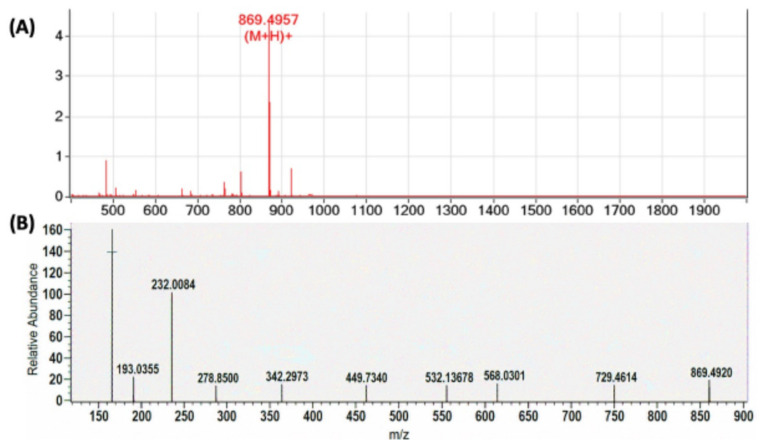
ESI-Q-TOF MS and MS/MS data for SP34. (**A**) ESI-Q-TOF MS spectrum of SP34, (**B**) MS/MS spectrum of SP34.

According to an HPLC chromatogram, SP34 had a purity of 90.47% at a retention time of 17.59 min, as shown in Figure 4. The chemical formula of SP34 is C_51_H_68_N_2_O_10_, with an average molecular weight of 869.1090 Da, and it was tentatively analyzed using the ESI-Q-TOF MS/MS data (Figure 5A,B).

### 2.4. Structural Elucidation Based on NMR Analysis

**Figure 6:**
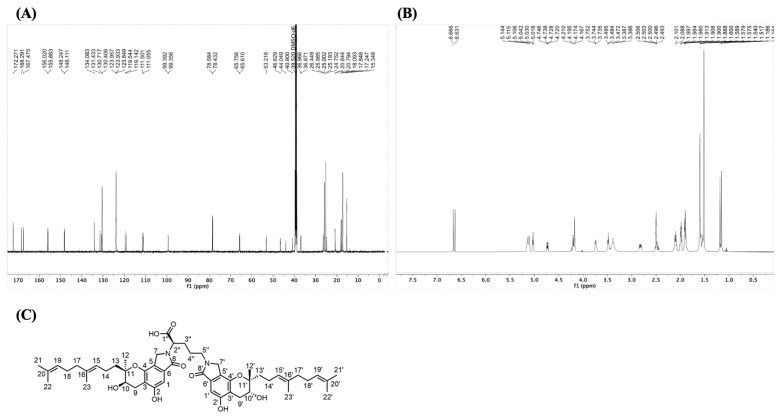
^1^H NMR and ^13^C NMR spectroscopic data of SP34. (**A**) ^13^C NMR spectroscopic data of SP34. (**B**) ^1^H-NMR spectroscopic data of SP34. (**C**) Stereochemical configuration of SP34.

^1^H NMR (DMSO-*d*_6_, 600 MHz) shows two peaks at δ 6.68 and 6.65, characteristic of aromatic or olefinic protons (Figure 6A). Two characteristic peaks at δ 5.15 and δ 5.04 are assigned to methylene and hydroxyl groups. Likewise, peaks at δ 4.75, δ 4.27–4.17, δ 3.75 and 3.50 correspond to oxymethylene (–O–CH_2_–), oxygenated methine (–CH–O–), and methoxy groups (–O–CH_3_), respectively. The aliphatic region contains multiple peaks indicating methylene and methine signals at δ 2.88–2.80 (m), 2.56–2.47 (m), and 2.18–2.05 (m).

The ^13^C NMR (DMSO-d_6_, 150 MHz) spectrum showing three peaks at δ 172.99, 169.01, and 168.19 corresponds to the carboxylic acid or a conjugated ester, as depicted in Table 2. Carbon signals at δ 100.11 and 100.07 represent 1-benzene (CH), and signals at δ 79.30, 79.15 (CH–O), δ 66.47, 66.32 (CH_2_–O), and δ 53.93 (possibly a methoxy carbon) indicate oxygenated carbon resonances (Figure 6B). The regions at δ 124.67, 124.65, 124.56, 120.26, and 119.86 and δ 112.22, 111.77, 100.11, and 100.07 correspond to aromatic CH groups and anomeric and other aromatic CH, respectively. The aliphatic carbons (δ 79–16) include upfield methylene and methine signals. Analysis of NMR and high-resolution mass spectrometry data together revealed that SP34 is a bisindole compound (Figure 6C), which was named Pandanicoline. However, the structure of this compound is consistent with the known chemical structure of FGFC1 [13].

### 2.5. Inhibition Rate of Pandanicoline on Na^+^-K^+^-ATPase and Ca^2+^-Mg^2+^-ATPase

**Figure 7:**
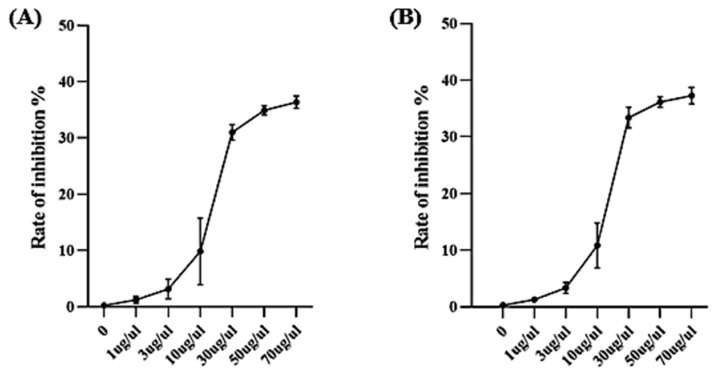
Inhibition rate of Pandanicoline on Na^+^-K^+^-ATPase and Ca^2+^-Mg^2+^-ATPase. (**A**) Inhibition rate of Pandanicoline on Na^+^-K^+^-ATPase. (**B**) Inhibition rate of Pandanicoline on Ca^2+^-Mg^2+^-ATPase. Comparisons among groups were carried out by one-way analysis of variance (ANOVA). Data are presented as mean ± standard deviation (SD). Each experimental condition was tested in at least three independent replicates (*n* = 3).

The inhibition rate of Na^+^-K^+^-ATPase by Pandanicoline increased ina Pandanicoline-concentration-dependent manner. At a concentration of 30 μg/μL, the inhibition rate reached its maximum; thereafter, as the Pandanicoline concentration continued to increase, the inhibition rate of Na^+^-K^+^-ATPase by Pandanicoline stabilized, with a maximum inhibition rate of 36.37% (Figure 7A). Likewise, the inhibition rate of Ca^2+^-Mg^2+^-ATPase reached a maximum of 37.27% at a concentration of 30 μg/μL (Figure 7B). When the Pandanicoline concentration increased to 50 μg/μL and 70 μg/μL, the inhibition rates of Na^+^-K^+^-ATPase and Ca^2+^-Mg^2+^-ATPase no longer increased. This is likely due to substrate depletion, preventing further reaction, and thus, the inhibition rates of Na^+^-K^+^-ATPase and Ca^2+^-Mg^2+^-ATPase plateau.

### 2.6. Effect on the Activity of Na^+^-K^+^-ATPase in HEK293 Cells

**Figure 8:**
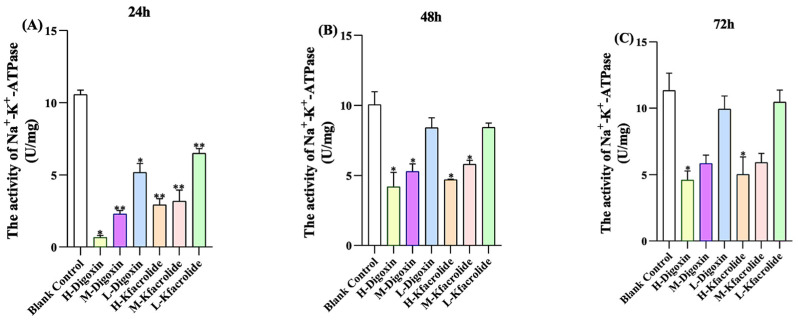
Activity of Na^+^-K^+^-ATPase in HEK293 cells. (**A**) Activity of Na^+^-K^+^-ATPase in HEK293 cells 24 h after drug administration. (**B**) Activity of Na^+^-K^+^-ATPase in HEK293 cells 48 h after drug administration. (**C**) Activity of Na^+^-K^+^-ATPase in HEK293 cells 72 h after drug administration. Comparisons among groups were carried out by one-way analysis of variance (ANOVA). Data are presented as mean ± standard deviation (SD). Each experimental condition was tested in at least three independent replicates (*n* = 3). Error bars in all graphs represent the standard deviation (SD). Values of * (*p* < 0.05); and ** (*p* < 0.01) were considered statistically significant.

To evaluate the effect of the identified bioactive compound on Na^+^-K^+^-ATPase activity, HEK293 cells were treated with different concentrations of Pandanicoline. As depicted in Figure 8, after 24 h, 48 h, and 72 h of Pandanicoline treatment in HEK293 cells, the activity of Na^+^-K^+^-ATPase in the control group increased with prolonged culture duration. Compared with the control group, the positive control at a concentration of 0.0625 mg/mL significantly attenuates the activity of Na^+^-K^+^-ATPase in HEK293 cells, with the most pronounced inhibitory effect observed. Moreover, as digoxin concentrations decreased, the inhibitory effect on the activity of Na^+^-K^+^-ATPase markedly diminished [14]. Similarly, the activity of Na^+^-K^+^-ATPase treated with Pandanicoline was also reduced, showing a trend consistent with positive digoxin. However, at equivalent concentrations, positive digoxin exhibited a stronger inhibitory effect on the activity of Na^+^-K^+^-ATPase than Pandanicoline.

### 2.7. Effect on the Activity of Ca^2+^-Mg^2+^-ATPase in HEK293 Cells

**Figure 9:**
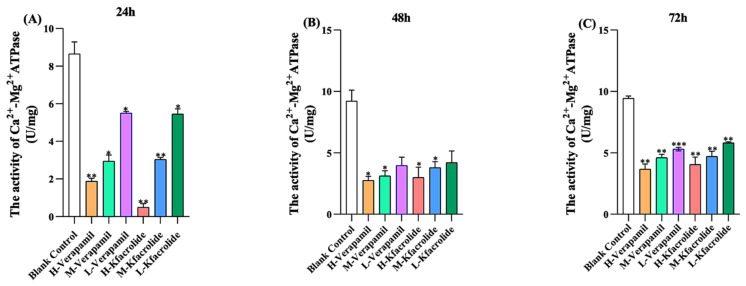
Activity of Ca^2+^-Mg^2+^-ATPase in HEK293 cells. (**A**) Activity of Ca^2+^-Mg^2+^-ATPase in HEK293 cells 24 h after drug administration. (**B**) Activity of Ca^2+^-Mg^2+^-ATPase in HEK293 cells 48 h after drug administration. (**C**) Activity of Ca^2+^-Mg^2+^-ATPase in HEK293 cells 72 h after drug administration. Comparisons among groups were carried out by one-way analysis of variance (ANOVA). Data are presented as mean ± standard deviation (SD). Each experimental condition was tested in at least three independent replicates (*n* = 3). Error bars in all graphs represent the standard deviation (SD). Values of * (*p* < 0.05); and ** (*p* < 0.01); *** (*p* < 0.001) were considered statistically significant.

**Figure 10:**
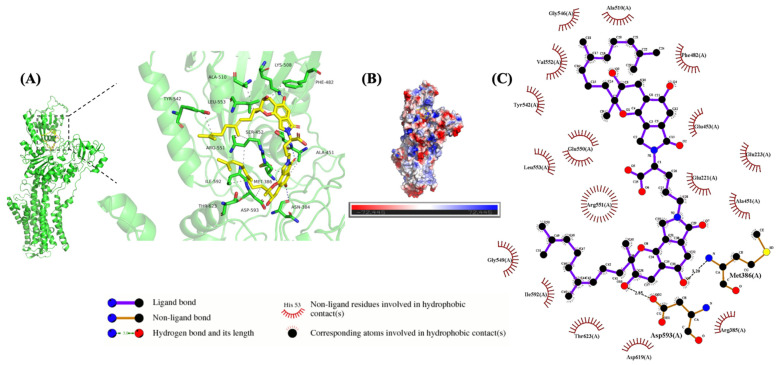
Schematic representation of the interaction between Pandanicoline and Na^+^-K^+^-ATPase. (**A**) The green regions represent Na^+^-K^+^-ATPase, and the yellow regions represent Pandanicoline. The blue dashed lines indicate hydrogen bonds, and the gray dashed lines indicate hydrophobic interactions. (**B**) Potential diagram of the interaction between Pandanicoline and Na^+^-K^+^-ATPase. (**C**) 2D diagram of the interaction between Pandanicoline and Na^+^-K^+^-ATPase.

Likewise, after 24 h of Pandanicoline treatment, compared with the blank control group, the activity of Ca^2+^-Mg^2+^-ATPase decreased in HEK293 cells treated with positive verapamil and Pandanicoline, as shown in Figure 9. Its activity decreased as drug concentration increased, with the activity of Ca^2+^-Mg^2+^-ATPase diminishing. At a positive drug concentration of verapamil (0.0625 mg/mL), the inhibitory effect was most pronounced. The activity of Ca^2+^-Mg^2+^-ATPase in the blank control group increased with prolonged culture time up to 72 h (Figure 10C), while the positive drug verapamil and Pandanicoline consistently inhibited the activity of Ca^2+^-Mg^2+^-ATPase.

### 2.8. Molecular Docking of Pandanicoline to Na^+^-K^+^-ATPase

The binding pocket for Pandanicoline with Na^+^-K^+^-ATPase is centered on the coordinates (124.732, 110.833, 42.678), which correspond to the centers of the ATP-binding residue 487 and the catalytic residue 376 in UniProt. The binding energy of Pandanicoline with Na^+^-K^+^-ATPase was −9.124 kcal/mol. The compounds primarily bind to each other through hydrogen bonding and hydrophobic interactions [15]. Pandanicoline formed hydrogen bonds with amino acids, especially ASN384, MET386, ALA451, SER452, and LYS508 (Figure 10A–C). The Pandanicoline formed hydrophobic interactions with amino acids including SER452, PHE482, LYS487, ALA510, ARG551, LEU553, ILE592, ASP593, and THR623.

### 2.9. Molecular Docking of Pandanicoline to Ca^2+^-Mg^2+^-ATPase

**Figure 11:**
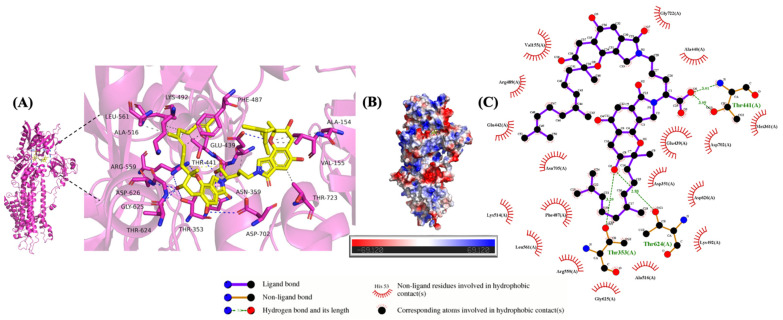
Schematic representation of the interaction between Pandanicoline and Ca^2+^-Mg^2+^-ATPase. (**A**) The pink regions represent Ca^2+^-Mg^2+^-ATPase, and the yellow regions represent Pandanicoline. The yellow dashed lines indicate salt bridges, the gray dashed lines indicate hydrophobic interactions, and the blue dashed lines indicate hydrogen bonds. (**B**) Potential diagram of the interaction between Pandanicoline and Ca^2+^-Mg^2+^-ATPase. (**C**) 2D diagram of the interaction between Pandanicoline and Ca^2+^-Mg^2+^-ATPase.

Pandanicoline bound to the Ca^2+^-Mg^2+^-ATPase at the center of the pocket (−39.733, −60.314, −19.631). The binding affinity of Pandanicoline with the Ca^2+^-Mg^2+^-ATPase complex was −10.47 kcal/mol. They primarily interact through hydrogen bonds and hydrophobic interactions [16]. In Figure 11A–C, the Pandanicoline formed hydrogen bonds with the amino acids THR353, ASN359, GLU439, THR441, ARG559, THR624, GLY625, and ASP626. The Pandanicoline also formed a salt bridge with the amino acid ARG559. The Pandanicoline-forming hydrophobic interactions with amino acids were ALA154, VAL155, THR723, THR353, LEU561, ALA516, LYS492, PHE487, and GLU439.

## 3. Discussion

The current study identifies a marine-derived *Sirastachys pandanicola* ZSDH2536 as a source of a Pandanicoline-based regulator of Na^+^-K^+^-ATPase and Ca^2+^-Mg^2+^-ATPase, two enzymes central to ionic homeostasis and electromechanical signaling [17]. Systematic screening revealed that secondary metabolites of ZSDH2536 attenuate the activity of both enzymes, indicating the presence of a selective inhibitory metabolite rather than nonspecific metabolic interference. Na^+^-K^+^-ATPase and Ca^2+^-Mg^2+^-ATPase coordinate intracellular Na^+^ and Ca^2+^ dynamics, thereby regulating membrane potential, excitation–contraction coupling, vesicular trafficking, and signal transduction [18]. The dual inhibitory profile observed here is therefore notable, as coordinated suppression of Na^+^ extrusion and Ca^2+^ handling has direct implications for cellular excitability and contractility [19].

Molecular identification based on 18S rRNA sequencing, phylogenetic reconstruction, and detailed morphological and ultrastructural analyses confirmed that ZSDH2536 grouped with *Stachybotrys longispora*. Marine-derived *Sirastachys pandanicola* is increasingly recognized for producing chemically diverse bioactive metabolites, exhibiting various pharmacological properties, especially enzyme- and membrane-targeting activities. ATPase inhibition observed in this study aligns with previous results demonstrating marine fungal polyketides as regulators of ion transport systems [20]. The secondary metabolites were tentatively identified in the ZSDH2536 strain, and a known compound was identified based on ^1^H-NMR, ^13^C-NMR, and high-resolution mass spectrometry and named Pandanicoline. The chemical formula of Pandanicoline is C_51_H_68_N_2_O_10_, with an average molecular weight of 869.1090 Da (isotopic mass 868.4874 Da). When Pandanicoline was assessed against Na^+^-K^+^-ATPase and Ca^2+^-Mg^2+^-ATPase activity rate, at 30 μg/μL, Pandanicoline attenuated the Na^+^-K^+^-ATPase rate by 36.37% and Ca^2+^-Mg^2+^-ATPase by 37.27%. At 50 μg/μL and 70 μg/μL, the inhibitory effects of Pandanicoline on Na^+^-K^+^-ATPase and Ca^2+^-Mg^2+^-ATPase reached a plateau, with no further increases observed. This saturation likely resulted from substrate depletion, which halted any additional enzymatic progression. Functional assays in HEK293 cells demonstrated that Pandanicoline inhibited the activities of Na^+^-K^+^-ATPase and Ca^2+^-Mg^2+^-ATPase in a concentration- and time-dependent manner. Although digoxin and verapamil exhibited greater potency, Pandanicoline reproduced the same inhibitory effects. Importantly, the progressive increase in ATPase activity in untreated controls confirms the specificity of Pandanicoline-mediated inhibition rather than generalized cytostatic effects [21].

Molecular docking analyses revealed that Pandanicoline bound to Na^+^-K^+^-ATPase and Ca^2+^-Mg^2+^-ATPase with binding energies of −9.124 kcal/mol (hydrogen bonding with key amino acids: ASN384, MET386, ALA451, SER452, and LYS508) and −10.47 kcal/mol (hydrogen bonding with THR353, ASN359, GLU439, THR441, ARG559, THR624, GLY625, and ASP626), respectively. These values suggest that Pandanicoline meets the structural and energetic requirements for stable, specific interactions with both target enzymes at the computational level [13,14]. In summary, these findings indicated that Pandanicoline derived from marine *Sirastachys pandanicola* ZSDH2536 is an ATPase inhibitor with a distinct chemical scaffold, supporting the broader concept that marine fungi constitute an underexplored reservoir of ion-transport modulators with translational relevance. Future studies should address isoform selectivity, binding kinetics, and efficacy to define its therapeutic scope in vivo. Previously, the Pandanicoline drug exhibited significant and selective antitumor activity, primarily targeting EGFR-mutated non-small cell lung cancer (NSCLC) cells and drug-resistant acute myeloid leukemia (AML) cells by elevating intracellular reactive oxygen species (ROS) levels and inhibiting key signaling pathways, such as PI3K/Akt/mTOR and NF-κB, thereby triggering apoptosis, pyroptosis, and cell cycle arrest (G0/G1 phase) [22,23,24].

## 4. Materials and Methods

### 4.1. Procurement of Materials

Sea mud collected from Zhoushan, Zhejiang Province, was stored in a −80 °C refrigerator. Potatoes purchased from Dingdong Shopping, Shanghai, China. Glucose (AR), sucrose (AR), starch soluble (AR), beef paste peptone (BR), NaNO_3_ (AR), K_2_HPO_4_ (AR), NaCl (AR), NH_4_Cl (AR), MgSO_4_ (AR), KCl (AR), CoCl_2_ (AR), FeSO_4_ (AR), CaCl_2_ (AR), phosphoric acid (AR), HCl (AR), NaOH (AR) (Sinopharm Chemical Reagent Co., Ltd., Shanghai, China); agar (Haibo Biotechnology Co., Ltd., Qingdao, China); L-ornithine hydrochloride (BR), physiological saline (Shanghai yuanye Bio-Technology Co., Ltd., Shanghai, China); defatted soybean flour (AR) (Shanghai Shifeng Biotechnology Co., Ltd., Shanghai, China); bacteriological peptone (BR) (Huankai Microbial Technology Co., Ltd., Guangzhou, China); yeast extract (BR) (Shanghai Thermo Fisher Scientific Technology Co., Ltd., Shanghai, China); CHCl_3_ (AR) (Shanghai Lingfeng Chemical Reagent Co., Ltd., Shanghai, China); anhydrous alcohol (AR), ethyl acetate (AR), methanol (AR), glutaraldehyde (AR), osmium acid (AR), tert butanol (AR), methanol (HPLC), acetonitrile (HPLC), TFA (HPLC) (Shanghai Titan Technology Co., Ltd., Shanghai, China); minim ATP enzyme test kit (Beijing Baiao Leibo Technology Co., Ltd., Beijing, China); TSINGKE Plant DNA Extraction Kit (Universal) (Tsingke Biotechnology Co., Ltd., Beijing, China); PBS (Wuhan Saiweier Co., Ltd., Wuhan, China).

### 4.2. Preliminary Crude Extraction of Marine Strains

The fermentation solution of the strain was centrifuged at 8000 r/min for 15 min, and the supernatant was discarded to retain the precipitate. The mixture was thoroughly mixed with anhydrous methanol, and the extraction was assisted by ultrasound 3 times (1 min each time). After centrifugation again (8000 r/min, 15 min), the precipitate was discarded, and the supernatant was concentrated. The concentrate was stratified using trichloromethane, and then the top oil layer was poured out and set aside. The water layer (pH = 3) was adjusted with a 1% aqueous solution of phosphoric acid, and it was extracted three times with ethyl acetate. After that, 5% anhydrous sodium sulfate was used to remove the water to obtain the ethyl acetate phase. We concentrated the upper oil layer into a semi-solid; afterwards, the anhydrous methanol was dissolved. Finally, the methanol phase was obtained. The ethyl acetate and methanol phases were centrifuged, filtered through a 0.22 μm organic phase filter membrane, and then stored in a 4 °C refrigerator pending measurement.

### 4.3. Na^+^-K^+^-ATPase Assay

Screening methods for the inhibition activity of Na^+^-K^+^-ATPase of crude extractions consisted of two parts: the enzymatic reaction and the determination of phosphorus content. This process was divided into a test group, control group, blank group and standard group [25].

$$Enzyme\;activity=[(\frac{{OD}_{test}-{OD}_{Control}}{{OD}_{standard}}-{OD}_{blank})\times 0.02\frac{\mu mol}{mL}\times 6\times 7.8\times C_{p}$$

For the test group, 0.12 mL of double-distilled water, 0.1 mL of the sample (microbial crude extracts mixed with liver tissue dilution, 5:5, *v*/*v*), 0.04 mL of reagent X, and 0.42 mL of matrix solution (reagents I, II, and III in a 260:80:80 ratio) were sequentially added to a centrifuge tube. The mixture was incubated at 37 °C for 10 min, after which 0.1 mL of reagent IV was added. Following thorough mixing, the tube was centrifuged at 3500 rpm again for 10 min, and the supernatant was retained for phosphate measurement.

For the control group, we added 0.16 mL of double-distilled water and 0.42 mL of matrix solution (reagent I: reagent II: reagent III = 260:80:80) to a centrifuge tube. We mixed the reagents and placed the reaction in an incubator at 37 °C for 10 min. We then added 0.1 mL of reagent IV and 0.1 mL of the sample and mixed thoroughly, centrifuged them at 3500 r/min for 10 min, and took the supernatant. Phosphorus content of the control group was determined by the same reaction method as that of the test group. The average of the parallel groups (*n* = 3) was calculated.

In the blank group, we mixed 0.3 mL of double-distilled water with 1.0 mL of color developer thoroughly and left it at room temperature for 2 min. After that, it was mixed with 1.0 mL of reagent VI and stood again at room temperature for 5 min. After it was zeroed with double-distilled water at 636 nm, the absorbance values were measured. The average value of the parallel group (*n* = 3) was calculated.

In the standard group, 0.3 mL of phosphorus standard solution (0.02 μmol/mL) was mixed thoroughly with 1.0 mL color developer and left to stand for 2 min at room temperature. After that, it was mixed with 1.0 mL of reagent VI and stood at room temperature for 5 min. It was zeroed at 636 nm with double-distilled water, and the absorbance values were determined. The mean values of parallel groups (*n* = 3) were calculated.

The results were expressed in units of ATPase activity, defined as the amount of inorganic phosphate (1 μmol) produced per hour per milligram of protein by ATPase hydrolysis, denoted as (U/mg protein).

### 4.4. Ca^2+^-Mg^2+^-ATPase Assay

The first step is an enzymatic reaction. We added 0.1 mL of the sample (deionized water: liver tissue dilution = 5:5), 0.08 mL of reagent VIII, 0.08 mL of reagent IX, and 0.42 mL of matrix solution (reagent I:reagent II:reagent III = 260:80:80), in turn, to a centrifuge tube. The above reagents were mixed and placed in an incubator at 37 °C for 10 min. They were mixed thoroughly with 0.1 mL of reagent IV. Then, they were centrifuged at 3500 r/min for 10 min, and the supernatant was taken to determine phosphorus [12].

The determination methods for the test group, control group, blank group, and standard group of Ca^2+^-Mg^2+^-ATPase are the same as those described in Section 4.3. Results were expressed in units of ATPase activity, defined as the amount of inorganic phosphate (1 μmol) produced per hour per milligram of protein by ATPase hydrolysis, denoted as (U/mg protein).

### 4.5. Strains Culture Methods

In this experiment, the strains were isolated from sea mud, which was collected from Zhoushan, Zhejiang Province (longitude 122.3500, latitude 30.0179). The strains were cultured on PDA medium (potato juice 20%, glucose 2%, agar 2%) for 5 days at 28 °C. After 5 days, we chose an inoculated ring of the strains and inoculated them in GSP medium (glucose 3.5%, starch soluble 1%, defatted soybean flour 2%, bacteriological peptone 0.5%, beef paste peptone 0.5%, yeast extract 0.3%, NaCl 0.2%, K_2_HPO_4_ 0.05%, MgSO_4_ 0.005%, pH 5.8) in a shaker (ZWYC-2932; Shanghai Zhicheng Analytical Instrument Manufacturing Co., Ltd., Shanghai, China) at 28 °C and 180 r/min for 3 days. Aspirated 5% GSP strains were cultured in fermentation medium (sucrose 0.05%, NaNO_3_ 0.3%, K_2_HPO_4_ 0.05%, MgSO_4_ 0.005%, KCl 0.005%, *Saccharomyces cerevisiae* extract 0.1%, CoCl_2_ 0.25‰, FeSO_4_ 1.5‰, CaCl_2_ 0.65‰, L-ornithine hydrochloride 1%, pH 5.8) at 28 °C and 180 r/min in a shaker (ZWYC-2932; Shanghai Zhicheng Analytical Instrument Manufacturing Co., Ltd., Shanghai, China) for 5 days. The fermentation solution of the strain was obtained.

### 4.6. Strain Identification

The marine fungi were sent to Shanghai Sangong Bioengineering Co., Ltd., Shanghai, China, for strain determination. The 18S rDNA-ITS sequences of fungi were amplified using fungal universal primers (ITS 1: TCCGTAGGTGAACCTGCGG, ITS 4: TCCTCCGCTTATTGATATGC) as upstream and downstream primer sequences [26]. We uploaded the ITS rDNA sequence of marine microorganism FG126 to NCBI, downloaded 20 gene sequences similar to ZSDH2536, and performed sequence alignment. We constructed a phylogenetic tree using the Neighbor-Joining Method in the MEGA 12.0.14 software.

### 4.7. Scanning Electron Microscopy

The colony morphology of marine microorganism ZSDH2536 was examined under a Hitachi Regulus SU8100 Scanning Electron Microscope (Hitachi High-Technologies Corporation, Tokyo, Japan). Sample preparation method for scanning electron microscopy of marine microorganism ZSDH2536: We placed a cover slip at an angle on the surface of the PDA solid medium used to cultivate marine microorganism ZSDH2536, allowing the fungus to naturally climb onto the cover slip. After cultivation, we removed the coverslip coated with marine microorganism ZSDH2536 and immersed it in a 2.5% glutaraldehyde fixation solution. It was fixed at 4 °C for 2 h; washed three times with PBS (phosphate-buffered saline) for 10 min each time; and immersed in 1% osmium acid solution at 4 °C for 2 h. It was dehydrated sequentially with 50%, 70%, 95%, and 100% ethanol (including two rounds of 100% ethanol dehydration), each for 10 min. The sample underwent tert-butanol substitution, freeze-drying, mounting, gold-coating, and examination under the microscope [27].

### 4.8. Preparation of Secondary Metabolites from Marine Microorganism ZSDH2536

**Figure 12:**
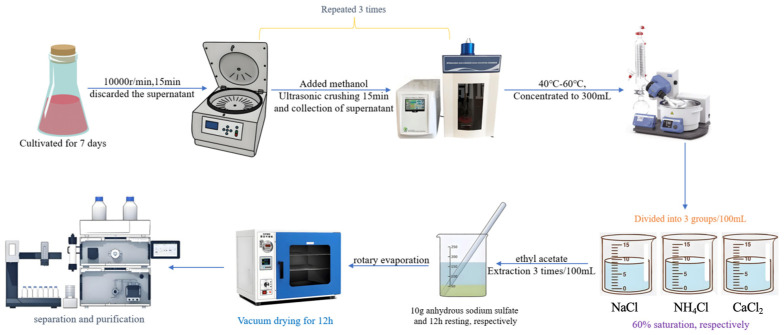
Process for the preparation of secondary metabolites from marine microorganism ZSDH2536.

At the end of the 7-day fermentation, an equal volume of methanol was added to each conical flask, followed by ultrasonic extraction for 15 min. The mixture was then centrifuged at 10,000 r/min for 15 min, and the precipitate was discarded. The supernatant was concentrated to 300 mL under reduced pressure at 40–60 °C with the removal of methanol. The pH of the concentrated solution was adjusted to 3.0 and divided into three groups (100 mL each). Sodium chloride, ammonium chloride, and calcium chloride were added separately to each group to achieve 60% saturation. The resulting mixtures were extracted three times with ethyl acetate (100 mL each). The ethyl acetate layers were combined, dried over anhydrous sodium sulfate (10 g) for 12 h, filtered, and concentrated to dryness under reduced pressure. The residue was further dried under vacuum for 12 h, dissolved in a minimal amount of methanol, and subjected to HPLC analysis, as shown in Figure 12.

### 4.9. HPLC Analysis

The analytical HPLC equipment was a Waters 1525 system consisting of a Waters 1525 binary pump, a Waters 2487 UV–vis photodiode array detector, a Waters 2707 injection valve with a 20 µL loop, and a Waters HPLC workstation (Waters, Milford, MA, USA). The column applied in this work was an XTerra MS C18 column (250 mm × 4.6 mm, 5 µm, Waters, USA). The system ran with a gradient program at 1 mL/min and two solvents, acetonitrile (A) and water (B), with the following gradient combinations: 0–10 min, 30% B; 10–20 min, 30–60% B; 20–25 min, 60–90% B; 25–30 min, 90–60% B. The eluent was monitored at 254 nm, and the purity was calculated by the target analyte peak area divided by the total peak area (unitary area method).

### 4.10. Separation and Identification of Metabolites in ZSDH2536

The combination of analytical HPLC and pre-HPLC procedures resulted in the successful fractionation of the sample into five chromatographically resolved fractions. The sample components underwent rotary evaporation at 40–60 °C for volume reduction, followed by lyophilization in a freeze–drying system. The sample components were dissolved in HPLC-grade methanol at a concentration range of 0.5–1 mg/mL. The chromatographic analysis was performed under the following optimized conditions: column temperature maintained at 35 °C, detection wavelength set at 255 nm, an injection volume of 5 μL, and a mobile phase flow rate controlled at 1.0 mL/min. The final mobile phase conditions for LC-MS/MS analysis were optimized as follows: initial condition: 10% acetonitrile (ACN) with 90% aqueous phase (0.1% TFA in H_2_O) maintained for 0–20 min. A linear gradient was applied to transition to 50% ACN with a 50% aqueous phase (0.1% TFA in H_2_O) over a 0–20 min period. The composition was returned to 10% ACN with a 90% aqueous phase (0.1% TFA in H_2_O) from 40 to 60 min. The final equilibration step maintained the 10% ACN condition for 60.1–65 min.

The mass spectrometric analysis was conducted using an ESI-Q-Exactive Orbitrap mass spectrometer (Thermo Fisher Scientific INC., San Jose, CA, USA) under optimized conditions using positive and negative ion source mode detection. The gas flow parameters were carefully regulated, with a sheath gas flow rate of 5, a sweep gas flow rate of 0, and an auxiliary gas flow rate of 0 (arbitrary units). Temperature control was achieved by stabilizing the interface temperature at 300 °C and maintaining the desolvation temperature at 526 °C. Full-scan mass analysis was performed across the *m*/*z* range of 20–1000 to ensure comprehensive detection of analytes. For the MS/MS, three different stepped normalized collision energies (20 and 30) were applied with the same instrument to perform the MS/MS experiments. Operating conditions for the instrument were as follows: sheath gas flow rate of 10, auxiliary gas flow rate of 0 (arbitrary units), spray voltage of 3.50 kV, capillary temperature of 320 °C, and S-lens Rf level of 50. All experimental conditions were strictly controlled to maintain method reproducibility and analytical sensitivity.

Metabolite analysis of the acquired mass spectrometric data was performed using Progenesis QI (version 3.0, Nonlinear Dynamics, UK) [16]. The software performed chromatographic alignment by selecting a reference run and correcting retention time drifts across all samples using alignment vectors. Feature detection (peak picking) was carried out using an absolute ion intensity threshold of 5000. Protonated [M+H]^+^ and sodiated [M+Na]^+^ adducts were included in the workflow for compound identification in positive ionization mode [17]. The processed features were subsequently matched against the MassBank of North America (MoNA) and ChemSpider databases for tentative identification. Features were evaluated based on precursor ion accuracy (≤5 ppm), fragmentation pattern similarity, and isotopic distribution and classified according to the Schymanski level of identification [16]. Additionally, tentative compound identification was further achieved by identifying accurate mass, fragmentation patterns, and isotope distribution with online databases, including MoNA, METLIN, and FoodB [18].

### 4.11. NMR Analyses

In addition, the purified compound was concentrated to dryness under reduced pressure and lyophilized, followed by dissolution in deuterated DMSO for NMR analysis. The ^1^H and ^13^C spectra were obtained on a Bruker Avance III 600 MHz NMR spectrometer (Bruker Biospin Co., Billerica, MA, Rheinstetten, Germany). ^1^H-nuclear magnetic resonance (NMR) and ^13^C-NMR spectra were obtained at the Center of Analysis, Shanghai Microspectrum Chemical Technology Service Co. Ltd. (Shanghai, China).

### 4.12. Na^+^-K^+^-ATPase and Ca^2+^-Mg^2+^-ATPase Inhibition Assay

The activities of Na^+^-K^+^-ATPase and Ca^2+^-Mg^2+^-ATPase were measured separately according to the methods described in Section 4.3 and Section 4.4. Pandanicoline was added at concentrations of 0 μg/μL, 1 μg/μL, 3 μg/μL, 10 μg/μL, 30 μg/μL, 50 μg/μL and 70 μg/μL, and the inhibition rates of activities of Na^+^-K^+^-ATPase and Ca^2+^-Mg^2+^-ATPase at different concentrations of Pandanicoline were determined [28].

$$Rate\;of\;Inhibition\left(\%\right)=\left[\frac{A_{0}-A_{t}}{A_{0}}\right]\times 100$$

*A*_0_ is enzyme activity without Pandanicoline; *A_t_* is enzyme activity with Pandanicoline added.

### 4.13. In Vitro Experiment

Human Embryonic Kidney 293 Cells were provided by Biyuntian Biotechnology Co., Ltd., Shanghai, China, and cultured as per the standard protocol. The HEK293 cells were cultured in minimum essential medium (Shanghai Beckman Biological Technology Co., Ltd., Shanghai, China) supplemented with 10% (*v*/*v*) fetal bovine serum (FBS) (Wuhan Saiweier Co., Ltd., Wuhan, China) and 1% (*v*/*v*) penicillin–streptomycin (Shanghai Meisen Biotechnology Co., Ltd., Shanghai, China) at 37 °C under 5% CO_2_ in a humidified incubator (Thermo Fisher Scientific, Waltham, MA, USA), with medium changed every 48–72 h until confluence. Upon reaching 80–90% confluence, HEK293 cells were passaged using 0.25% trypsin-EDTA (Shanghai Meisen Biotechnology Co., Ltd., China). HEK293 cells at passages 4–7 were used for subsequent experiments [29].

When the HKE293 cells reached 80–90% confluence, the culture medium was aspirated. Digoxin (Shanghai Titan Technology Co., Ltd., China), verapamil (Shanghai Titan Technology Co., Ltd., China), or Pandanicoline was added to each well at final concentrations of 0.0625, 0.03125, and 0.015625 mg/mL (2 mL per well). The control group received 2 mL of drug-free medium. Each condition was performed in triplicate. Cells were then incubated at 37 °C in a humidified atmosphere containing 5% CO_2_ for 24, 48, or 72 h. At the indicated time points, the drug-containing medium was removed, and cells were harvested by trypsinization, followed by centrifugation (Hitachi Limited Co., Ltd., Tokyo, Japan). After discarding the supernatant, cell pellets were resuspended in 200 µL of normal saline (Hiseano Health Technology Co., Ltd., Qingdao, China) and lysed by sonication (Hitachi Limited Co., Ltd., Japan) (3 cycles, 1 min each).

### 4.14. Molecular Docking

The AutoDockTools software (version 1.5.7) was used to perform docking simulations for Pandanicoline, with Na^+^-K^+^-ATPase and Ca^2+^-Mg^2+^-ATPase. The three-dimensional structures of Na^+^-K^+^-ATPase and Ca^2+^-Mg^2+^-ATPase were obtained from the Protein Data Bank (accession numbers P05023 and P16615, respectively). Pandanicoline was visualized and edited by ChemOffice Professional 23 and converted into a 3D structure using the ChemBio 3D module. The optimal molecular docking conformation was determined via the “Iterated Local Search” algorithm in the molecular docking program Auto Dock Vina (Version 1.2.3) [30,31,32]. The data analysis and visualization were displayed by using the PyMOL (Version 3.0.3) and Maestro (Version 13.9.135).

### 4.15. Statistical Analysis

Data were analyzed using GraphPad Prism 8.0.2, and results were expressed as mean ± SEM by one-way analysis of variance (ANOVA). Values of * *p* < 0.05 were considered statistically significant.

## 5. Conclusions

A novel bidirectional bioactivity assessment system was established to screen microbial metabolites for inhibitory effects on the activities of Na^+^-K^+^-ATPase or Ca^2+^-Mg^2+^-ATPase. The secondary metabolites of strain ZSDH2536 exhibited a significant inhibitory effect on the activities of Na^+^-K^+^-ATPase and Ca^2+^-Mg^2+^-ATPase. The current investigation reveals marine-derived *Sirastachys pandanicola* ZSDH2536 as a potent source of structurally distinct ATPase regulators, and it is non-toxic. HRMS and NMR helped in identifying a known compound of alkaloids called Pandanicoline (C_51_H_68_N_2_O_10_) from marine-derived *Sirastachys pandanicola* ZSDH2536 for the first time. Pandanicoline demonstrates concentration- and time-dependent reductions in the activities of Na^+^-K^+^-ATPase and Ca^2+^-Mg^2+^-ATPase in HEK293 cells. Pandanicoline increases its structural ability for ATPase-targeted intervention while potentially offering improved safety profiles. The binding energies for the molecular docking of Pandanicoline with Na^+^-K^+^-ATPase and Ca^2+^-Mg^2+^-ATPase were −9.124 kcal/mol and −10.47 kcal/mol, respectively. This indicates that Pandanicoline has a structural and energy basis to form stable and specific binding with target proteins at the computational level and is a key step towards becoming a “lead compound” in the drug discovery process. However, future investigations should focus on isoform-specific binding mechanisms, in vivo efficacy in disease models, and nutraceutical applications. Pandanicoline thus represents a promising molecule for developing safer, mechanistically distinct therapeutics targeting a fundamental ionic regulatory pathway.

## Figures and Tables

**Table 1:** The morphology of marine microorganisms in different culture media.

NO.	Name	PDA Medium	GSP Medium	Czapek Agar Medium
(1)	YM Y-5-5	Spherical	Spherical	Globular
(2)	F720	Slender white mycelium	Sticky state	Flocculation
(3)	R2006	Spherical	Spherical	Globular
(4)	F11193	White filamentous mycelium	Viscous state	white flocculent
(5)	VCS-95	Spherical	Spherical	Globular
(6)	TW1-170	Spherical	Coccus	Coccoid
(7)	FD180	Black spherical	Light red globular	Light red globular
(8)	white	White short mycelium	Thick	Flocculent state
(9)	ZSDH2536	Villous to arachnoid	Flocculate	Flocculate

**Table 2:** ^1^H NMR and ^13^C NMR data of compound SP34 in DMSO-*d*_6_.

No.	δ ^1^H (ppm)	δ ^13^C (ppm)
1	6.68 or 6.65	100.11 or 100.07
2		156.74 or 156.60
3		112.22 or 111.77
4		148.96 or 148.83
5		120.26 or 119.86
6		132.15 or 131.43
7	4.22	44.76
8		169.01
9	2.84 or 2.51	27.16
10	3.75	66.47 or 65.32
11		79.30 or 79.15
12	1.18	18.81 or 18.57
13	1.59	37.68 or 37.59
14	2.12	21.56 or 21.51
15	5.14	124.67 or 124.65
16		134.82 or 134.80
17	1.92	40.00
18	2.00	26.70 or 26.62
19	5.05	124.56
20		131.12
21	1.60	25.91
22	1.53	17.96
23	1.53	16.06
1′	6.68 or 6.65	100.11 or 100.07
1″		172.99
10′	3.75	66.47 or 65.32
11′		79.30 or 79.15
12′	1.18	18.81 or 18.57
13′	1.59	37.68 or 37.59
14′	2.12	21.56 or 21.51
15′	5.14	124.67 or 124.65
16′		134.82 or 134.80
17′	1.92	40.24
18′	2.00	26.70 or 26.62
19′	5.05	124.56
2′		156.74 or 156.60
2″	4.75	53.93
20′		131.12
21′	1.60	25.91
22′	1.53	17.96
23′	1.53	16.06
3′		112.22 or 111.77
3″	2.00	26.62
4′		148.96 or 148.83
4″	1.55	25.47
5′		120.26 or 119.86
5″	3.50	41.61
6′		132.15 or 131.43
7′	4.22	47.35
8′		168.19
9′	2.84 or 2.51	27.16
DMSO_d6	3.50	40.00

## Data Availability

The raw data supporting the conclusions of this article will be made available by the corresponding authors upon request.
